# O8-3 A school-based quasi-experimental intervention to improve students' motor competence and physical fitness

**DOI:** 10.1093/eurpub/ckac094.059

**Published:** 2022-08-29

**Authors:** Mikko Huhtiniemi, Kasper Salin, Timo Jaakkola

**Affiliations:** Faculty of Sport and Health Sciences, University of Jyväskylä, Jyväskylä, Finland

**Keywords:** school-aged, intervention, fitness, motor competence

## Abstract

**Background:**

Previous research has shown that school can be an influential context to promote students' physical activity engagement, physical fitness, and motor competence. The purpose of this study was to investigate the effectiveness of a 5-month-long intervention program that aimed to increase students' motor competence and physical fitness during school days.

**Methods:**

A quasi-experimental intervention design with pre- and post-tests was implemented. Altogether 325 Finnish Grade 5 (Mage = 11.26, *SD* = .33) students from five schools participated in the study. At the beginning of the study, students were divided into experimental and control groups on purpose. The intervention consisted of three components: a) weekly 20 minutes sessions of guided training during a regular PE lesson, b) weekly 20 minutes sessions of guided training during recess and c) daily 5 minutes long activity breaks during academic lessons. Intervention activities aimed to increase different elements of physical fitness and motor competence. One week before and one week after the intervention period students' physical fitness levels were measured by 20-meter shuttle run (cardiorespiratory fitness), curl-up and push-up (muscular fitness) tests, and motor competence by 5-leaps (locomotor skills) and throwing-catching-combination (object control skills) tests.

**Results:**

Repeated measures MANOVA indicated that there was a multivariate interaction effect between experimental and control groups over time (F [5, 222] = 7.52, p=.000, partial η^2^=.145). Moreover, univariate analysis revealed that students in the experimental group performed significantly better in 20-meter shuttle run test (F [1, 226] = 21,9, p=.000, partial η^2^=.088), curl-up (F [1, 226] = 4,9, p=.028, partial η^2^=.021), push-up (F [1, 226] = 15,5, p=.000, partial η^2^=.064) and throwing-catching-combination (F [1, 226] = 4,0, p=.046, partial η^2^=.017). There were no differences between experimental and control groups in 5-leaps test (F [1, 226] = 0.003 p=.958, partial η^2^=.000).

**Conclusions:**

The intervention program appeared to be effective in increasing students' cardiorespiratory fitness, muscular fitness, and object control skills. This indicates that guided school-based physical activity programs can be influential in promoting physical fitness and motor competence among school children.

